# Deemed consent for organ donation in Northern Ireland

**DOI:** 10.1016/j.lanepe.2021.100254

**Published:** 2021-11-04

**Authors:** Jordan A. Parsons, Bonnie Venter

**Affiliations:** a Centre for Ethics in Medicine, Bristol Medical School, University of Bristol, UK; b Centre for Health, Law, and Society, Bristol Law School, University of Bristol, UK

## Abstract

Shortages of organs for transplantation have led many countries to introduce systems of deemed consent for organ donation, whereby donation is the default upon death and an individual must provide express opposition to donation to prevent it. Despite a lack of clear supporting evidence, it is often suggested that deemed consent will contribute significantly to addressing the organ shortage. Northern Ireland appears set to be one of the next countries to pursue this route, with the Organ and Tissue Donation (Deemed Consent) Bill currently making its way through the Northern Ireland Assembly. If passed, this Bill will see Northern Ireland follow in the footsteps of the rest of the UK. In this viewpoint, we provide an overview of Northern Ireland's progress towards introducing deemed consent and argue two related points. First, that public awareness of the policy (if introduced) is vital to both its defensibility and longevity, and that this must be recognised through the imposition of a ministerial duty to focus on such awareness. Second, that policymakers in Northern Ireland ought to support the policy to ensure consistency across the UK in organ procurement, thereby preventing Northern Ireland from disproportionately benefitting from the UK-wide organ allocation system.

Systems of deemed consent for organ donation – also known as presumed consent or opt out – make the donation of certain bodily material the default upon death, requiring an expressed opposition to prevent procurement proceeding. This approach generally rests on the understanding that more individuals are willing to donate than are registered donors under an expressed consent system [1]. Given the global shortage of organs for transplantation, interest has grown in the deemed consent model and its perceived potential for increasing transplantation activity. Indeed, moratoria on many transplantation programmes during the COVID-19 pandemic [2,3] have increased the need for transplantable organs. Countries with both deemed consent systems and high transplantation rates, such as Spain, are often looked to in evidencing the promise of deemed consent [4]. However, it is important to recognise that the success of the Spanish system is more a result of investment in education and organisation, such as increasing the number of transplant coordinators and placing them inside hospitals, and fostering good working relationships with the mass media [5,6]. Indeed, it was not until these organisational changes came 10 years after the introduction of deemed consent that deceased donation activity in Spain began to markedly increase. It has been suggested that Spain's deemed consent legislation is ‘dormant’ as a result [7].

One of the most recent countries to move in the direction of deemed consent is Northern Ireland. In this viewpoint, we provide an overview of Northern Ireland's progress towards introducing deemed consent and argue two related points. First, that public awareness of the policy (if introduced) is vital to both its defensibility and longevity, and that this must be recognised through the imposition of a ministerial duty to focus on such awareness. Second, that policymakers in Northern Ireland ought to support the policy to ensure consistency across the UK in organ procurement, thereby preventing Northern Ireland from disproportionately benefitting from the UK-wide organ allocation system.

## 1 Changes in Northern Ireland

Following the ‘encouraging’ (though thus far unpublished) results of a public consultation which closed in February 2021 [8,9], the Organ and Tissue Donation (Deemed Consent) Bill was laid before the Northern Ireland Assembly in July 2021 – it has since passed its Second Stage and will now proceed to Committee Stage. This is not the first attempt to introduce deemed consent in Northern Ireland, with a 2015 Bill failing at the committee stage due to divided committee opinion [10]. Nonetheless, whilst the new Bill's journey through the Assembly has just begun, it is reasonable to assume that it will eventually be enacted, even if in a slightly amended form, given the public consultation – the earlier, failed Bill did not follow such a consultation. This is largely because the public consultation acts as an exercise in bringing the public into the policy process and, where concerns raised are appropriately responded to in the development of the eventual law, garnering public support. Indeed, whilst many of the concerns raised about deemed consent will be consistent across countries, there may be some that are particular to the national context which may result in a slightly different model to those existing elsewhere at the time. The process of public consultation also acts as a first step in public awareness of any eventual policy change which, as we will come to highlight, is of great importance with deemed consent for organ donation.

If enacted, the Bill will amend the Human Tissue Act 2004 (in relation only to activities carried out in Northern Ireland) and introduce a system largely similar to those established throughout Great Britain in recent years [11,12]. Proposed amendments to section 3 of the Human Tissue Act 2004 are identical to those made by the Organ Donation (Deemed Consent) Act 2019 in England: in the absence of an expressed decision by the deceased adult in force immediately before their death, their consent to organ donation will be deemed unless ‘a person who stood in a qualifying relationship to the person concerned immediately before death provides information that would lead a reasonable person to conclude that the person concerned would not have consented’. The policy will be applicable only to adults – children will remain under the purview of the existing expressed consent system.

What will constitute sufficient information to conclude that the deceased would not have consented will, as elsewhere in the UK, fall to eventual clinical guidance. In Great Britain, there has been some variation on this point. Whereas guidance in England and Scotland states that donation ought *not* to proceed where no one in a qualifying relationship is contactable [13,14], Welsh guidance permits donation to proceed in such circumstances provided certain requirements are met [15]. It will be interesting to see where the Northern Ireland Assembly will place itself on this point; just how “deemed” will the country's deemed consent be.

In line with the rest of the UK, Northern Ireland's proposed system will allow for ‘excepted adults’. Excepted adults will include those who have not been ordinarily resident in Northern Ireland for the 12 months immediately prior to their death and those who lacked capacity to understand the implications of the policy for a significant period before their death. ‘Excepted adults’ will continue to fall under the expressed consent system.

## 2 Public awareness duties

Notably, the Northern Irish Bill proposes to impose a duty on the Department of Health in relation to public awareness of the new system. This will come in the form of amendments to the Health (Miscellaneous Provisions) Act (Northern Ireland) 2016, which already imposes a duty to promote transplantation and annual information campaign efforts. The proposed amendments will more specifically impose a duty of annual efforts to make the public aware of the impact of the deemed consent system and how those who desire to can opt out.

Such a duty acknowledges the significance of deeming consent in contrast with the current system of expressed consent, and how this sits with ethico-legal conceptions of informed consent generally. Per the landmark UK Supreme Court case of *Montgomery* [16], informed consent requires that the patient be made aware of ‘any reasonable alternative or variant treatments’ and of material risks that they might attach significance to. Whilst the statutory introduction of deemed consent means that informed consent per *Montgomery* would not apply, it is interesting to reflect on how the two compare – at the very least as a consideration of potential public perception. In deceased organ donation where expressed consent applies, informed consent can be understood to require that the deceased was aware of the options both to donate and to not, and what their decision would entail, for the consent to be valid. For the consent to be *deemed*, then, a comparable principle requires that the patient was aware of the system and what their *in*action would entail.

A case in the European Court of Human Rights concerning the transplantation of a man's organs in Latvia opined that ‘reasonable enquiries’ should be made to try and understand what the deceased would have decided [17]. This idea is clearly encapsulated in the existing deemed consent laws in the UK, whereby the family are consulted ahead of donation. However, in this case the Court did not opine that donation should not proceed where such ‘reasonable enquiries’ fail to prove informative, which might be taken as permitting a system closer to so-called ‘hard’ deemed consent than is ordinarily proposed. Nonetheless, mapping deemed consent onto the principles of informed consent demonstrates that the requirement of sufficient public awareness is to, in effect, constitute knowledge of material risks and ‘reasonable alternatives’. ‘Reasonable enquiries’ are then to be made, with the family providing an almost proxy confirmation. The ethical literature similarly comments that the defensibility of deemed consent rests on citizens being aware of the system and it not being unreasonably difficult for them to opt out [18]. Hence the importance of the public awareness duty in preventing consent to organ donation being ‘deemed’ inappropriately.

Interestingly, the Northern Irish Bill proposes that this duty rest on the Department of Health. The existing deemed consent systems of Wales and Scotland place a similar duty on Welsh and Scottish Ministers, respectively. Whilst this may be seen as much the same thing in practice, given that the Northern Irish Department of Health is led by Northern Irish Ministers, it may be considered slightly broader in introducing a collective responsibility. Equally, however, one might consider this a slight blame avoidance tactic. Nonetheless, that a duty exists at all is to be viewed positively as the Northern Ireland Executive recognising the high stakes of the proposed policy change and assuming responsibility in line with ethico-legal expectations.

Whilst the proposed Northern Ireland model in relation to public awareness duties is appropriate – much like those in Wales and Scotland, even if slightly different – it raises an important consideration when thinking about the existing deemed consent system in England. Something of an outlier, the Organ Donation (Deemed Consent) Act 2019 in England does *not* impose a duty to make public awareness efforts [12]. There have been indications that such efforts are intended [19,20], but a statutory duty has not been established [21]. What makes this especially noteworthy is that the Welsh system – which does include such a duty – was in place for several years before the enactment of the Organ Donation (Deemed Consent) Act 2019. There was, therefore, an active departure from this earlier example, suggesting less importance was placed on ethico-legal expectations in England. This is a problematic aspect of the English system which ought to be avoided both by Northern Ireland – which seems set to be the case – and any other country introducing a system of deemed consent in future. A statutory duty not only introduces accountability for public awareness, but also sends an important principled message that policy makers take seriously the potential pitfalls of deemed consent regarding public support.

There are many simple actions that enable ministers to execute this duty regarding public awareness. For example, inclusion of organ donation as a compulsory element of school curricula – something which NHS Blood and Transplant provides teaching resources to support [22]. Not only can this contribute to greater awareness in younger generations, but it may also spill over into older generations by children asking family members about it outside of school. The precise approaches to public awareness planned for Northern Ireland if the Bill is passed are as yet unclear. However, the accompanying Explanatory and Financial Memorandum does indicate an anticipated annual expenditure of £4-500,000 for nine years, to cover ‘public education and awareness, change management, IT infrastructure changes, processing additional registrations, evaluation and clinical training’ [23]. This is perhaps optimistically low, particularly since the Welsh Government spent £3.2m in the three financial years starting 2013/14 [24], but it is too early to assess the public awareness efforts of Northern Ireland.

## 3 The need for intra-UK consistency

Whilst the Welsh system has seen some success with increases in public awareness [25] and consent rates [26], there are reasons to be sceptical of the ability of deemed consent to address the organ shortage [27,28]. However, the success of the policy against that measure is not central to our discussion. Whether deemed consent will improve transplantation rates or not, we suggest that there is a strong ‘justice as fairness’ [29] argument for politicians in Northern Ireland to support the Bill and see it into law. This stems from the present disconnect between organ procurement and allocation in the UK. Organ allocation is organised at the UK level, with all four nations participating in a standard protocol, even though it does have in-built elements of “local” prioritisation. As such, an organ donated in one nation might be transplanted into a patient in another. As long as Northern Ireland operates an expressed consent model, recipients in Northern Ireland might be considered to disproportionately benefit when receiving an organ donated in Great Britain through deemed consent. If deemed consent does increase organ donation, Northern Ireland may be viewed as what game theory deems the ‘free rider’, benefitting from the collective action of Great Britain without contributing and, in doing so, avoiding the potential issues associated with deemed consent. For example, in this scenario Northern Ireland would not risk the change prompting public mistrust in the healthcare system, nor would it have to shoulder the financial cost of switching to the new policy.

Here, we adopt Rawls’ ‘veil of ignorance’ approach [29], whereby one pursues the fairest option from a position of lack of knowledge of one's position in society. Where the different parties all have the means to contribute in the same way (contribution meaning alignment on the policy of deemed consent rather than number of transplantable organs), it can be concluded that equal benefit should be afforded where there is equal contribution. As such, to benefit from the shared allocation system, Northern Ireland ought to contribute on broadly the same (policy) terms. This strict equality approach ought only to be diverged from where there are pertinent inequalities in ability to contribute. If Northern Ireland did not have the means to implement deemed consent, then, it would be appropriate to allow this potential for disproportionate benefit – equity rather than equality. This is, however, not the case. Hence, Northern Ireland ought to align with the rest of the UK in implementing deemed consent, even if it does little to improve transplantation rates.

## 4 Conclusion

Now that transplantation programmes are resuming and the world is beginning to emerge from COVID-19 lockdowns, attention on means of improving the availability of transplantable organs will inevitably begin to increase once more. No doubt, systems of deemed consent will continue to be considered by policymakers, and existing examples will be looked to for both evidence and inspiration. Where this happens, it is vital that the ethico-legal aspects of deemed consent are suitably accounted for which, we contend, requires the imposition of a ministerial duty to regularly raise public awareness of the implications of deemed consent. In Northern Ireland specifically, we call on politicians to support the present Bill to ensure consistency across the UK, thereby pursuing a justice as fairness approach to this matter of health policy.

## Contributors

JAP wrote the first draft of the manuscript. Both authors contributed to critical revision of the manuscript. Both authors read and approved the final manuscript.

## Declaration of Interests

Both authors declare no competing interests.
